# Patient Perspectives on Conversational Artificial Intelligence for Atrial Fibrillation Self-Management: Qualitative Analysis

**DOI:** 10.2196/64325

**Published:** 2025-03-12

**Authors:** Ritu Trivedi, Tim Shaw, Brodie Sheahen, Clara K Chow, Liliana Laranjo

**Affiliations:** 1 Westmead Applied Research Centre Faculty of Medicine and Health The University of Sydney Westmead Australia; 2 Charles Perkins Centre School of Medical Sciences The University of Sydney Sydney Australia; 3 Cardiology Department Westmead Hospital Westmead Australia

**Keywords:** atrial fibrillation, conversational agents, qualitative research, self-management, digital health, patient perspective, conversational artificial intelligence, speech recognition

## Abstract

**Background:**

Conversational artificial intelligence (AI) allows for engaging interactions, however, its acceptability, barriers, and enablers to support patients with atrial fibrillation (AF) are unknown.

**Objective:**

This work stems from the Coordinating Health care with AI–supported Technology for patients with AF (CHAT-AF) trial and aims to explore patient perspectives on receiving support from a conversational AI support program.

**Methods:**

Patients with AF recruited for a randomized controlled trial who received the intervention were approached for semistructured interviews using purposive sampling. The 6-month intervention consisted of fully automated conversational AI phone calls (with speech recognition and natural language processing) that assessed patient health and provided self-management support and education. Interviews were recorded, transcribed, and thematically analyzed.

**Results:**

We conducted 30 interviews (mean age 65.4, SD 11.9 years; 21/30, 70% male). Four themes were identified: (1) interaction with a voice-based conversational AI program (human-like interactions, restriction to prespecified responses, trustworthiness of hospital-delivered conversational AI); (2) engagement is influenced by the personalization of content, delivery mode, and frequency (tailoring to own health context, interest in novel information regarding health, overwhelmed with large volumes of information, flexibility provided by multichannel delivery); (3) improving access to AF care and information (continuity in support, enhancing access to health-related information); (4) empowering patients to better self-manage their AF (encouraging healthy habits through frequent reminders, reassurance from rhythm-monitoring devices).

**Conclusions:**

Although conversational AI was described as an engaging way to receive education and self-management support, improvements such as enhanced dialogue flexibility to allow for more naturally flowing conversations and tailoring to patient health context were also mentioned.

**Trial Registration:**

Australian New Zealand Clinical Trials Registry ACTRN12621000174886; https://tinyurl.com/3nn7tk72

**International Registered Report Identifier (IRRID):**

RR2-10.2196/34470

## Introduction

Atrial fibrillation (AF) prevalence is increasing rapidly [1], resulting in substantial costs to health systems [2]. AF care is multifaceted, and appropriate management is complex but key to preventing stroke, heart failure, and all-cause mortality [3]. Novel solutions to address these challenges and deliver integrated AF care are warranted [3]. Digital tools could be used to deliver self-management support and education to promote healthy lifestyle behaviors, medication adherence, symptom management, and overall ability to manage AF and prevent hospitalization [4]. However, existing digital interventions for patients with AF have shown limited engagement [5-7].

Conversational AI is a novel technology that can converse with users interactively by mimicking human-like conversations [8], but its feasibility in supporting AF patients is unknown. The evaluation of conversational AI has been limited, with the majority of studies being quasi-experimental and small-scale feasibility pilots exploring conversational AI for the prevention, diagnosis, and management of chronic conditions, but mostly for psychological conditions (depression and anxiety) and very few in cardiovascular diseases (heart failure, diabetes, and hypertension) [9-11]. Only a few randomized controlled trials (RCTs) have been conducted evaluating the effectiveness of conversational AI, revealing positive outcomes of improvements in symptoms of anxiety and depression in college students [12,13], insulin adherence and glycemic control in patients with type 2 diabetes [10], and medication adherence in patients with both or either hypertension and diabetes [11]. Notably, high engagement with users was common in these trials [10-13] and in other studies [9]. The application of conversational AI may provide a more engaging experience for patients with AF self-managing their condition.

Understanding user perspectives on digital health technologies is essential, especially in AF, as the condition manifests differently for all patients [14]. There is a paucity of qualitative research assessing the perspectives and experiences of patients with AF in using digital self-management interventions, with existing studies focusing on mobile apps [15-18] and none exploring conversational AI. Given the novelty of the technology, qualitative research is required to understand the user experience and acceptability of conversational AI to support AF self-management. Although an existing study has explored the feasibility of a conversational agent (non–AI-based) in patients with AF and has found high acceptance among users, it did not involve a formal qualitative analysis [15]. Qualitative research can help in understanding the barriers and enablers that influence AF patient engagement, which is important in narrowing the gap between proof-of-concept and implementation [19].

The Coordinating Health care with AI–supported Technology for patients with AF (CHAT-AF) [20] was a 6-month RCT evaluating a postdischarge digital program that comprised voice-based conversational AI (automated phone calls with speech recognition and natural language processing), text messages, emails, and an educational website to support patients with AF in their management journey. In this paper, we report on the qualitative findings from semistructured interviews postintervention assessing user perspectives, enablers, and barriers regarding the intervention.

## Methods

### Study Design

This qualitative study stems from a 6-month RCT, CHAT-AF, aimed at evaluating the feasibility of a conversational AI support program for patients with AF in the community [20]. This study adheres to the Consolidated criteria for Reporting Qualitative (COREQ) research checklist for reporting qualitative research.

### Ethical Considerations

The ethics committee at Western Sydney Local Health District (2020/ETH02546) granted approval and the study was registered with the Australian New Zealand Clinical Trials Registry (ACTRN12621000174886). All participants provided informed consent to take part in both the RCT and qualitative interviews. Data collected from this study was only accessible by approved study personnel and was stored on secure servers. Deidentification of data was done before analysis and publication.

### Setting and Participants

Participants were recruited for the RCT after discharge from inpatient or outpatient cardiology services at Westmead Hospital (Sydney, Australia). Adult AF patients competent with the English language who had access to a mobile phone were eligible. AF patients randomized to the intervention group were contacted via phone postintervention by study investigator RT and asked whether they would like to participate in a phone interview. These participants were chosen using maximum variation sampling, such that, people of different sex, age, ethnicity, education, type of AF, and engagement with the intervention were invited to take part in the interview.

### Intervention

**Figure 1:**
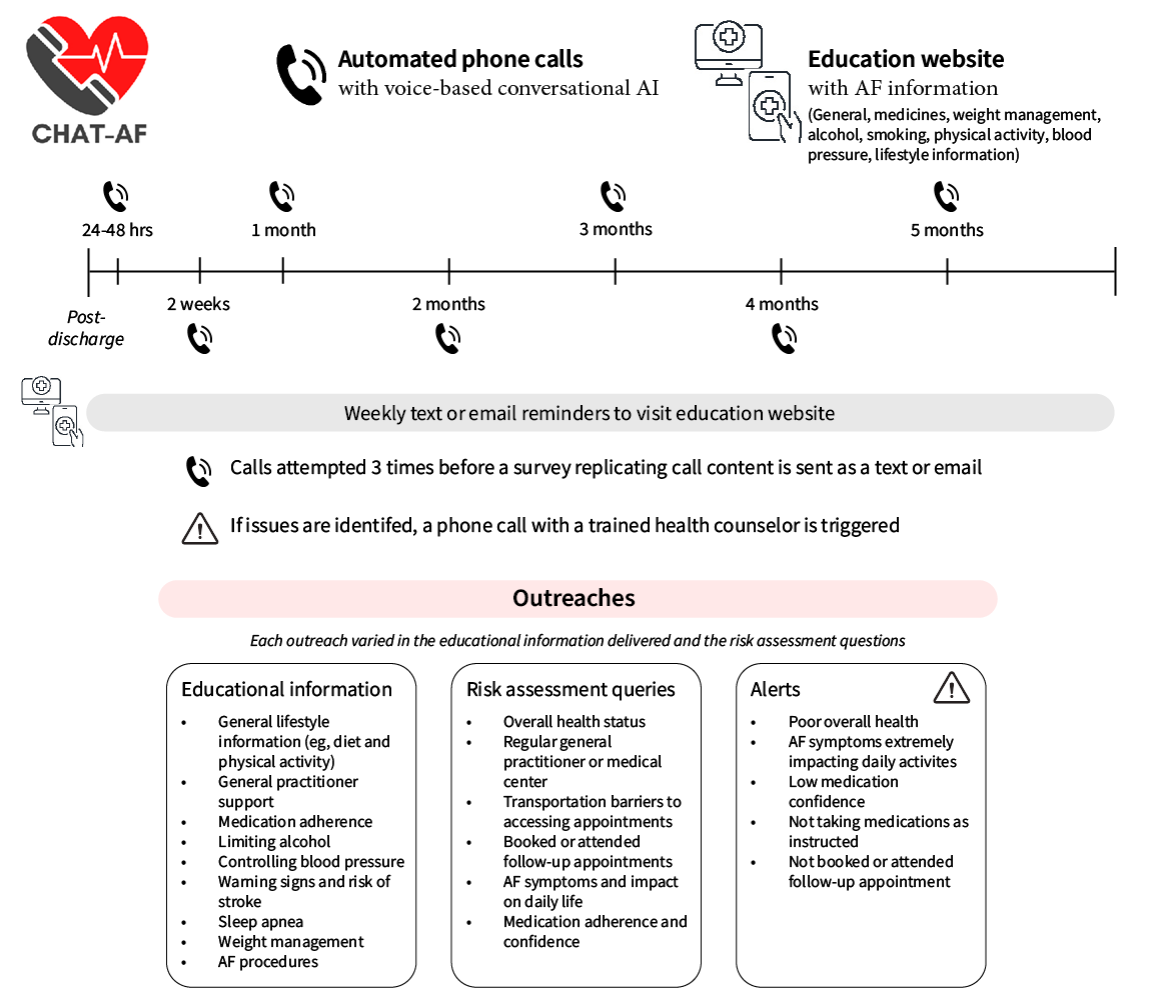
Coordinating Health care with Artificial intelligence–supported Technology for patients with Atrial Fibrillation (CHAT-AF) intervention. AF: atrial fibrillation; AI: artificial intelligence.

The intervention was designed to monitor patient health status and symptoms and provide self-management and education to AF patients in the community [20] (Figure 1). Briefly, it consisted of 7 outreaches via fully automated conversational AI phone calls, text messages, or email, supplemented with weekly nudges (text message or email) to visit the semipersonalized educational website (personalized according to smoking status, alcohol consumption, hypertension diagnosis, prescription of anticoagulants or warfarin specifically). During the phone calls patients received AF education and were required to verbally respond to risk assessment queries, with certain patient responses triggering an alert to the study team to be actioned and escalated if needed. If the participant was unable to be reached after 3 failed call attempts (including voicemails directed to an inbound phone number), a text message or email was sent directing them to a survey replicating the call content.

The AI aspect of the intervention included two main components: (1) speech recognition, which allowed the participant’s verbal responses to be recognized and then translated into text and (2) natural language processing, which analyzed the semantic and syntactic components of the user’s input. The tool was culturally adapted to include a voice with an Australian accent. If the participant’s response was unclear, the system would first repeat the question and then prompt the participant to press a button that corresponds to their answer (eg, “press 1 for always, 2 for often, and 3 for sometimes”). The system addressed participants by their first name and was programmed to refer to previous content areas, for example, if a participant had reported they had already visited their GP post their hospital discharge, it would not ask again.

### Modified Delivery of the Intervention

We had to prematurely stop recruitment in August 2021 due to the acquisition of the technology partner by another organization. At this point, there were 82 participants enrolled in the intervention arm, of these 20 had completed the intervention and 62 were at various stages of the program. All participants had received at least 2 of 7 outreaches via the conversational AI calls. The delivery of the intervention was then modified, and the call content was replicated and provided via survey only for the remaining outreaches.

### Data Collection Techniques

A semistructured interview approach was chosen to explore individual perspectives on conversational AI calls and other intervention components, along with exploring barriers and enablers to engagement with the intervention. The interview guide included open-ended questions and was developed based on existing literature reporting on the self-management needs of patients with AF and their perspectives on digital health interventions to support self-management [14-17] (Multimedia Appendix 1). The interview guide was pilot-tested within the research team. All interviews were conducted by the study investigator (RT) via phone and transcribed verbatim. Comprehensive analysis of interview content resulted in various iterations of themes and concepts. Interviews were conducted until sufficient depth was reached and no new themes, concepts, or relationships were emerging [21].

### Analysis

Interview transcripts were uploaded onto NVivo (QSR International) and thematically analyzed by 2 investigators (RT and LL). Coding was done inductively (first-cycle coding), followed by a grouping of codes based on thematic similarities and axial coding (ie, to find patterns between codes) [22]. The initial codebook was developed by RT, with discussions between investigators (RT and LL) occurring every 5 interviews to revise the codebook by analyzing emerging codes and themes. Refined versions of the codebook were reviewed by another investigator (BS) to ensure the appropriateness of focused and axial coding. Coauthor input (LL, BS, CKC, and TS) regarding themes and subthemes was gathered midway and at the completion of interviews.

## Results

**Figure 2:**
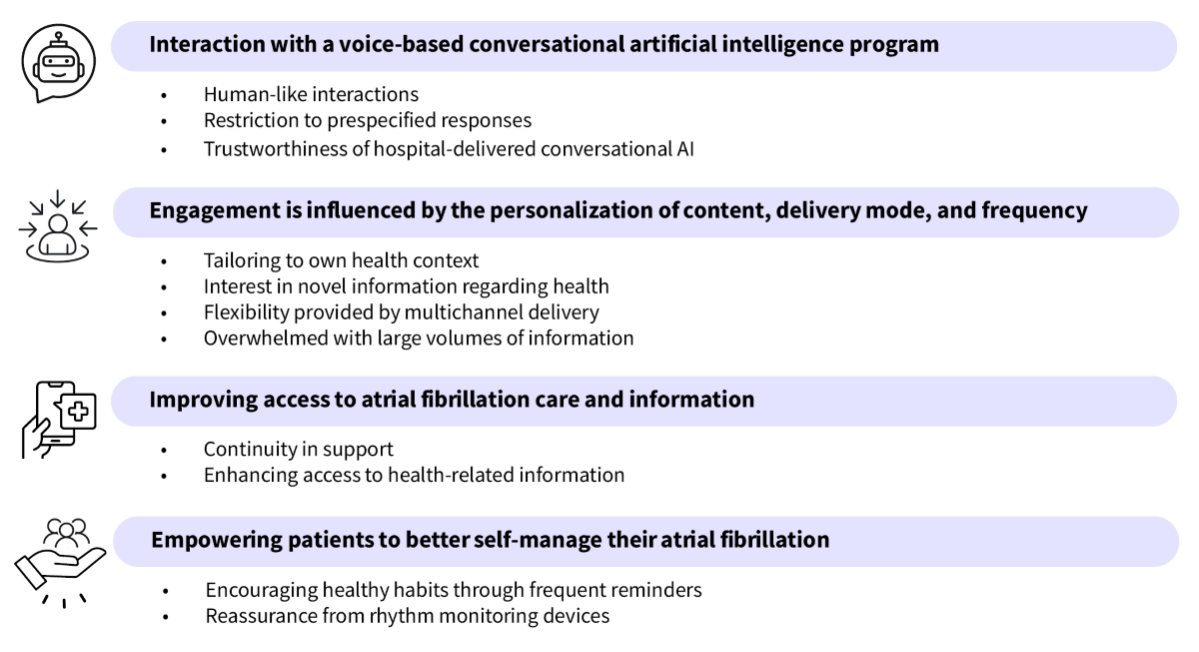
Themes and subthemes. AI: artificial intelligence.

We invited 33 intervention participants to this study and 3 were not interested in taking part in an interview. A total of 30 semistructured interviews (average duration of 20 minutes and 19 seconds) were conducted between August 2021 to May 2022. The mean participant age was 65.4 (11.91 SD) years, 70% (21/30) were male, 17% (5/30) were of non-Caucasian ethnicity, and 70% (21/30) were nonuniversity graduates (Table 1). Detailed characteristics of both the interviewed participants and the entire intervention cohort are available in Multimedia Appendix 2. Interviewed participants (n=30) received the full 6-month intervention, which included a total of 210 intervention outreaches: 154 were delivered via conversational AI and the remaining 54 were delivered only via the survey tool (modified intervention delivery mode). On average, 5.2 of 7 outreaches were delivered via conversational AI calls (original intervention delivery mode). Themes and subthemes are described below and summarized in Figure 2. Illustrative quotes for each theme are available in Multimedia Appendix 3.

**Table 1:** Participant characteristics and engagement metrics of the interviewed cohort (N=30 unless otherwise stated).

Characteristics		Values, n (%)
**Sex**		
	Male	21 (70)
	Female	9 (30)
**Age**		
	Mean (SD), years	65.4 (12)
	Younger than 64 years	13 (43)
	65-75 years	8 (27)
	Older than 75 years	9 (30)
**Ethnicity**		
	Caucasian	25 (83)
	Other Asian	2 (7)
	Chinese	1 (3)
	Arab or Persian	1 (3)
	Other	1 (3)
**Most recent atrial fibrillation type**		
	Paroxysmal	25 (83)
	Persistent	5 (17)
**Initial atrial fibrillation diagnosis (n=29)**		
	Less than 5 years ago	21 (70)
	5 years or longer	8 (27)
**Education**		
	Primary school	2 (7)
	Year 10 school certificate	5 (17)
	Year 12 higher school certificate	6 (20)
	Diploma/technical	8 (27)
	University graduate	9 (30)
**Annual household income, AUD^a^ (n=20)**		
	Less than $31,199	4 (13)
	$31,200-$77,999	9 (30)
	$78,000-$104,000	3 (10)
	More than $104,000	4 (13)
**Engagement with outreaches**		
	High (≥4 completed^b^)	25 (83)
	Low	5 (17)
**Engagement with website**		
	Visited once or less than once	19 (63)
Outreaches delivered via conversational artificial intelligence phone calls, of 7 total, mean (SD)		5.20 (1.81)

^a^The average conversion rate during the study was 1 AUD=US $0.72646.

^b^Participants answered ≥50% of questions asked in the outreach.

### Interaction With a Voice-Based Conversational AI Program

#### Human-Like Interactions

Participants reported that engaging with the phone calls felt “more human” and “personal” like “talking to a friend” (Multimedia Appendix 3, Table S1, quotes 1-5). Participants mentioned that the phone calls were interactive in the sense they provided a “two-way” channel of communication where they were being asked questions or given information and they were able to respond “easily” (Multimedia Appendix 3, Table S1, quote 5).

#### Restriction to Prespecified Responses

Participants mentioned the need for more dialogue flexibility, as they felt restricted by the predefined response options in the automated phone calls and wished they had an opportunity to elaborate on their answers (Multimedia Appendix 3, Table S1, quotes 6-8). They also mentioned that some questions did not apply to themselves and due to the lack of appropriate choices in response options, they were at times forced to choose an inappropriate answer, whereas a “does not apply” option would have prevented this issue (Multimedia Appendix 3, Table S1, quote 8).

#### Trustworthiness of Hospital-Delivered Conversational AI

The importance of the trustworthiness of conversational AI was evident as participants mentioned that bad experiences with marketing-based promotional calls could be a potential barrier to engagement (Multimedia Appendix 3, Table S1, quote 9). However, they highlighted that trusting the provider (ie, the hospital) created a sense of comfort when engaging with automated phone calls (Multimedia Appendix 3, Table S1, quote 10).

### Engagement Is Influenced by the Personalization of Content, Delivery Mode, and Frequency

#### Tailoring to Own Health Context

Participants mentioned that they would have found the program more useful and relevant if they were currently experiencing AF symptoms (Multimedia Appendix 3, Table S2, quote 1) and that they wanted targeted information that was relevant to their disease status (eg, time since diagnosis) and type of AF (eg, paroxysmal AF; Multimedia Appendix 3, Table S2, quotes 2 and 3). Participants noted that because their AF symptoms were not frequent, some questions did not apply to them (Multimedia Appendix 3, Table S2, quote 3).

Participants mentioned the program should be tailored based on their existing knowledge about the disease and their ability to self-manage (Multimedia Appendix 3, Table S2, quotes 4-9**).** Some thought the current content was probably more useful for recently diagnosed patients in greater need of information and support (Multimedia Appendix 3, Table S2, quotes 4 and 5) rather than people who had AF for a while (Multimedia Appendix 3, Table S2, quotes 6 and 7). The need for additional information about pacemakers (Multimedia Appendix 3, Table S2, quote 8) and the impact of COVID-19 on AF (Multimedia Appendix 3, Table S2, quote 9) was mentioned.

The need for tailored information based on an individual’s lifestyle behaviors was a recurring theme as participants felt that some of the lifestyle tips did not apply to them, for example, information about quitting smoking and limiting alcohol and caffeine (Multimedia Appendix 3, Table S2, quotes 10 and 11). It was also suggested that we include more comprehensive information about diet (Multimedia Appendix 3, Table S2, quote 12).

#### Interest in Novel Information Regarding Health

Participants mentioned they would have preferred more novelty in the content, with some of the information being similar to what is available from other reputable sources, such as the Heart Foundation (Multimedia Appendix 3, Table S2, quote 13 and 14). They also indicated the need for progressive content delivery to maintain engagement, for example, the weekly reminders to visit the education website were engaging but led to disappointment when participants realized there was no new content available (Multimedia Appendix 3, Table S2, quote 15).

#### Flexibility Provided by Multichannel Delivery

Participants differed in their preferences for communication. Some participants noted that it was easier to engage in phone calls and respond using their voice rather than scrolling on a survey, as they could complete the phone calls while multitasking, for example, going for a walk around the park (Multimedia Appendix 3, Table S2, quote 16). Some people also mentioned preferring to listen to information over a phone call, as they thought it was a more interactive and engaging process and they found it easier to interpret the information and visualize what had been said (Multimedia Appendix 3, Table S2, quotes 17-19). Others preferred to respond to the surveys instead of the phone calls, as surveys allowed for adequate time to think about questions before responding, could be completed at a convenient time, and were not interrupted by signal drops (Multimedia Appendix 3, Table S2, quotes 20-23).

#### Overwhelmed With Large Volumes of Information

Participants mentioned that the frequency of contact should also be tailored based on their needs and preferences, as some felt there were too many phone calls and reminders and would prefer to contact us on an “as needed” basis (Multimedia Appendix 3, Table S2, quotes 24 and 25). When asked about reasons for low intervention engagement, participants mentioned that they were dealing with other medical conditions that took precedence over their AF (Multimedia Appendix 3, Table S2, quote 26), and some participants did not want to be reminded of the problem as they were trying to stay positive (Multimedia Appendix 3, Table S2, quotes 26 and 27).

### Improving Access to Atrial Fibrillation Care and Information

#### Continuity in Support

Overall, participants saw the program as valuable in connecting them with care and providing important information and support (Multimedia Appendix 3, Table S3, quotes 1-8). Participants mentioned that emergency department visits were a “whirlwind experience” and “daunting,” and that there was typically no real support and education provided postdischarge, making them feel “helpless” (Multimedia Appendix 3, Table S3, quotes 2 and 3). Participants said that the program was able to address this gap by providing them with information and continuity in follow-up (Multimedia Appendix 3, Table S3, quotes 2-8)—“someone cares enough to contact you”—which was particularly important during COVID-19 when it was difficult to access doctors and for people needing social support (Multimedia Appendix 3, Table S3, quotes 5 and 6). Participants also highlighted the unpredictable and sporadic nature of AF and the need for continuous care as situations change quickly (Multimedia Appendix 3, Table S3, quote 7).

#### Enhancing Access to Health-Related Information

Participants mentioned that the program provided education and information about AF management that was difficult to obtain from clinicians due to multiple barriers, such as financial, geographical, and mobility constraints along with lack of time during consultations (Multimedia Appendix 3, Table S3, quotes 8-10). They also highlighted that information presented in clinical settings was often hard to absorb due to the high-stress environment and the use of complex terminology, and they appreciated receiving education in lay terms and in a simple format (Multimedia Appendix 3, Table S3, quotes 11 and 12). They also mentioned that the intervention was able to address existing knowledge gaps about AF and improve awareness (Multimedia Appendix 3, Table S3, quotes 13 and 14).

### Empowering Patients to Better Self-Manage Their AF

#### Encouraging Healthy Habits Through Health Reminders

Participants reported that the program improved their health behaviors as it served as a constant reminder and reinforcement of the need to follow healthy lifestyle habits and re-evaluate harmful ones (Multimedia Appendix 3, Table S4, quotes 1 and 2). Some participants mentioned that the program was an influencer in reducing their alcohol intake and promoting weight loss (Multimedia Appendix 3, Table S4, quotes 3 and 4). It was mentioned that the reminders were encouraging and motivational in prompting people to reflect on their current AF management, for example, remember to take medications, exercise, and focus on diet (Multimedia Appendix 3, Table S4, quotes 4-6).

#### Reassurance From Rhythm Monitoring Devices

The positives and opportunities for improvement of the conversational AI self-management intervention have been outlined in Textbox 1. Participants mentioned that having the ability to monitor their heart rhythm using consumer wireless devices or through an implanted device (loop recorder or pacemaker) can be reassuring in case they suspect the onset of AF and to better manage their symptoms and anxiety related to the disease (Multimedia Appendix 3, Table S4, quotes 7-10). Patients with implantable devices also mentioned that they would prefer being contacted more frequently to ensure that the device was working properly or having access to their own data to understand when things were normal and abnormal, as well as their individual triggers for AF (Multimedia Appendix 3, Table S4, quotes 11 and 12).

**Positives and opportunities for improvement regarding the use of a conversational AI self-management intervention for patients with atrial fibrillation.**

**Positive elements**

- Trustworthiness from receiving information from a hospital-linked provider.
- Engaging human-like interactions.
- Improved continuity in support.
- Improved access to health-related information.
- Frequent reminders and information.

**Areas for improvement**

- Improving dialogue flexibility of conversational AI phone calls to allow for more natural conversations.
- Enhancing personalization to one’s health context.
- Tailoring the amount of information to individual preferences.
- Reducing the repetitiveness of content.

## Discussion

### Principal Results

Our evaluation of a 6-month AF support program comprising voice-based conversational AI showed that participants valued being supported with their care needs outside of the clinical setting while indicating different barriers and enablers to engagement. Participants’ experiences with the conversational AI phone calls were generally positive, with some participants highly valuing that engagement method, and others being less keen on verbal communication for this purpose and preferring a text-based format. Overall, participants indicated the need for more dialogue flexibility, particularly in terms of their response options, to enable more engaging conversations. Personalization of the intervention to patients’ needs and preferences was also mentioned as key to engagement, with AF being an especially challenging disease given the high variability in patterns, symptoms, and clinical manifestations.

### Comparison With Previous Work

The current intervention was able to address the education and self-management needs of patients with AF, while also providing continuous care outside of a clinical setting. CHAT-AF participants appreciated receiving education and frequent self-care reminders, indicating the intervention addressed a gap in the provision of information and behavior change support. This gap had been previously described in other studies where AF patients expressed their desire for more information, assistance in creating healthy habits, and frequent motivational reminders [23,24]. In addition, it was appreciated that CHAT-AF enabled a greater ability to engage with content easily, when, and how the participant desired outside of a clinical environment. Knowledge gaps are prevalent in patients with AF [25] and patients have indicated insufficient opportunities for education during clinical visits [24]. Managing the ups and downs of the disease is a contributor to the poor quality of life experienced by patients [14] and could be addressed by providing additional support across the patient journey. Participants in our study appreciated the continuity in contact provided by the frequent digital “check-ups,” suggesting that interventions like CHAT-AF could enhance the transition between clinical care and community by providing a layer of interaction and support beyond traditional appointments.

Engagement with digital self-management programs by patients with AF has been suboptimal [5-7] but seems to be improved with conversational agents that can facilitate a human-like interaction[15]. A 1-month trial evaluating a mobile application with an embedded non-AI conversational agent showed high acceptability and engagement by patients (18 interactions over one month), even though the interactions required participants to touch prespecified options on the app, instead of using natural language [15]. The interactivity and convenience of voice-based conversational AI were mentioned by participants in CHAT-AF, and engagement was high over the 6-month intervention period, suggesting the value of such technology in supporting patients between clinical visits.

AF has a varied course, and patients appreciate the ability to freely express details regarding their AF rather than being confined to prespecified options, however, at present AI technologies do not allow for safe unconstrained interactions to deliver health information. Users of other AI-based conversational agents have also noted limitations in having naturally flowing conversations [12,13]. However, research has identified various safety concerns in unconstrained speech recognition of voice assistants in health care [26,27]. A study posing medical problems to popular conversational agents (eg, Alexa [Amazon], Google Assistant, and Siri [Apple Inc]), found that only 43% completed their given task, and of these completed tasks, 29% reported potentially harmful actions and 16% may have resulted in death [27]. Future developments in AI, specifically speech recognition and natural language processing, may be able to address the current dialogue shortcomings in our intervention, however, careful consideration is required for the safety implications of their use in health care.

Personalization was highlighted as a key factor to engagement, with challenges mentioned by AF patients given its episodic nature and sporadic symptoms leading to high variability in individual needs regarding education and self-management between different people and across time. AF patients often experience changes in their symptoms, triggers, and treatments, and their care should be reflective of these varying needs. Newly diagnosed AF patients will often require more education about AF [14], whereas those who have had AF for a prolonged time may have developed the skills to be able to self-manage but still require reinforcement of key concepts [24]. This highlights the need for adaptive health interventions that account for a change in patient needs throughout their disease and adapt accordingly, with the goal of maintaining engagement [19].

Trustworthiness was highlighted by participants as key to engagement with the AI intervention. With the rising popularity of chatbot-based search engines like the large language model ChatGPT, it is increasingly important to consider the factors that influence user trust in health information delivered via digital media [28]. Despite recent studies showing ChatGPT performs remarkably well in answering cardiovascular-related queries [29], its potential use as a health education tool is uncertain, given the lack of transparency in its sources of information. In our study, the credibility of the institution delivering the AI intervention was mentioned by participants as key in trusting the automated phone calls, given the proliferation of unsolicited and fraudulent “robocalls.” Patients’ acceptability of AI technology is still not well understood but seems to be higher when the intervention is recommended by clinicians [30,31].

### Implications

Programs like CHAT-AF could be used to deliver frequent education and self-management support between clinical visits to patients with AF at scale. In addition, conversational AI automated phone calls can improve access to care for traditionally disadvantaged AF patients in rural and remote communities [32], as phone calls are not reliant on internet connectivity, phone models, or operating systems. Engagement of digital interventions in less digitally literate populations remains challenging, however, the simplistic navigation of conversational AI interfaces (ie, requiring users to listen to information and respond using their voice) could provide utility in engaging with older adults who are most susceptible to AF alongside those with low technology literacy and should be the focus of future research. The findings of this work can also guide the implementation of other digital solutions for patients with AF, particularly regarding the optimization of interactivity and personalization, as well as the trustworthiness of providers and technology.

### Strengths and Limitations

This is the first qualitative study to explore the user perspectives of using conversational AI in supporting patients with AF. The premature study completion due to the acquisition of the technology partner by another organization meant that some participants had experienced fewer outreaches via conversational AI calls, which may have impacted how they reported on the AI component, however, all participants did receive a minimum of 2 outreaches (of 7) via the calls. As a single-site study, with a predominantly Caucasian and English-speaking population, generalizability is limited, and the results should be interpreted within the limitations of sample characteristics. For instance, the study population was younger (mean 65.4, SD 11.9 years) and had a lower proportion of women (30%) than previously reported literature of the AF patient population in Australia (mean age of 75 years and approximately 45% female) [33]. Selection bias may have occurred where those who agreed to the interviews may have been more engaged, interested, and motivated to provide feedback. We did not interview all trial participants and may have missed some unique experiences, although to address this, interviews were conducted until data saturation was reached. We did not involve patients in the design of the interview guide, and this may have resulted in not capturing experiences that are important to people with AF, however, we did prompt participants to share any additional comments regarding the research and intervention during the interview. Moreover, interview duration varied among participants and was on average around 20 minutes, it is possible that we may not have captured complex concepts in this time, however, the researcher used several prompts in the interview guide to facilitate further discussion during the interviews.

### Conclusions

This qualitative study of user experiences with a conversational AI program found that AF patients perceived it could address gaps in current models of AF care by improving access to information and providing continuity in support. Interactions with the voice-based conversational AI were perceived positively by participants, although the need for additional dialogue flexibility and response options was mentioned to better capture their AF experience. Future studies should explore whether delivering more personalized content and providing different interaction options can improve the engagement and effectiveness of such interventions.

## Appendix

Multimedia Appendix 1 Interview guide.

Multimedia Appendix 2 Characteristics of intervention participants and interview cohort.

Multimedia Appendix 3 Illustrative quotes for themes and subthemes.

## Abbreviations

AF: atrial fibrillation
AI: artificial intelligence
CHAT-AF: Coordinating Health care with AI–supported Technology for patients with AF
COREQ: Consolidated criteria for Reporting Qualitative
RCT: randomized controlled trial
