# Supplementary material for: Patient Perspectives on Conversational Artificial Intelligence for Atrial Fibrillation Self-Management: Qualitative Analysis
Source: J Med Internet Res. 2025 Mar 12;27:e64325. doi: 10.2196/64325 (PMC11947624; doi:10.2196/64325)
Supplement: Multimedia Appendix 1 [file jmir_v27i1e64325_app1.pdf]

Interview guide

What are some things that make it hard to live with atrial fibrillation and manage it?
What are some things that you do or use that make it easier to live with atrial fibrillation?
Do you currently use any technology to help with managing your health?
What were your thoughts about the program that you received?
What did you think when in the phone calls we would start off by asking about your overall health?
What did you think of the tips and information we presented you with in the phone calls?
How did you like the nature of the phone calls, the recorded voice talking to you?
What did you think of the voice that you were listening to over the phone?
How did you find the information on the education website?
We noticed that you were unable to complete some phone calls, could you expand on why? OR We noticed you couldn't get onto the education website, could you tell us more about why?
Part way through the program, did you notice that you stopped receiving phone calls and got text messages or emails instead. What did you think about this change?
Did you have any concerns about talking about your health over the phone or when completing the survey questions?
Would you have liked your general practitioner (GP) or other health professionals to be involved in this program?
Did you notice any change in how you managed your AF and health compared to before you started the program?
What would you like to see in future version of this program?
Is there something that is missing in your current AF care that you would like to see improved?

Consumer perspectives around conversational artificial intelligence (AI)
Did you think that you were talking to a person? Did it feel personal to you?
What did you think about the fact that you could response to the questions by just talking and it was able to recognise what you said?
Did you feel that it was able to understand you well?
Would have like to be able to talk more or have more response options?
How did you feel about listening to the robot-like voice giving you information about atrial fibrillation?
Did you find that you were able to understand the information? Would you say that you understood the information better over the phone rather than say if we were to give you a brochure?
Did you know that the robot is a type of AI technology? Have you heard of this before?
Knowing that this is a type of AI technology, does that affect your comfort level of engaging with the program?

Multi-channel communication
What did you think about the fact that we would contact you in different ways, such as, phone calls, text messages/emails?
Did you prefer a certain form of communication over the other, or did you like the combination of calls, messages, and email? If so, could you expand on why you thought that was the case?

Program supporting patients' needs
From your experience with this program, would you say that program that have these automated phone calls in which you listen to a human voice and answer by speaking can be useful to support patients like you?
Did you prefer to be contacted and receive information through a program like this one or would you refer to other websites, people and resources for support for your atrial fibrillation?
Do you think these programs may be useful to other patient groups? How do you think they fit into the bigger picture of supporting patients and their healthcare needs?
