# Supplementary material for: Patient Perspectives on Conversational Artificial Intelligence for Atrial Fibrillation Self-Management: Qualitative Analysis
Source: J Med Internet Res. 2025 Mar 12;27:e64325. doi: 10.2196/64325 (PMC11947624; doi:10.2196/64325)
Supplement: Multimedia Appendix 2 [file jmir_v27i1e64325_app2.pdf]

Characteristics of intervention participants and interview cohort

	Interview cohort, n (%)	Entire intervention cohort, n (%)
**Sex**	**N=30**	**N=82**
Male	21 (70.0%)	58 (70.7%)
Female	9 (30.0%)	24 (29.3%)
**Age**	**N=30**	**N=82**
Mean, years (SD)	65.4 (11.9)	63.8 (11.0)
< 64 years	13 (43.3%)	39 (47.6%)
65-75 years	8 (26.7%)	30 (36.6%)
> 75 years	9 (30.0%)	13 (15.9%)
**Ethnicity**	**N=30**	**N=81**
Caucasian	25 (83.3%)	60 (74.1%)
Other Asian	2 (6.7%)	4 (4.9%)
Chinese	1 (3.3%)	4 (4.9%)
Arab or Persian	1 (3.3%)	4 (4.9%)
Other	1 (3.3%)	9 (11.1%)
**Most recent atrial fibrillation type**	**N=30**	**N=82**
Paroxysmal	25 (83.3%)	64 (78.0%)
Persistent	5 (16.7%)	17 (20.7%)
Permanent/other	-	1 (1.2%)
**Initial atrial fibrillation diagnosis**	**N=29**	**N=78**
<5 years	21 (70.0%)	44 (56.4%)
≥5 years	8 (26.7%)	34 (43.6%)
**Education**	**N=30**	**N=81**
Primary school	2 (6.7%)	2 (2.5%)
Year 10 school certificate	5 (16.7%)	19 (23.5%)
Year 12 higher school certificate	6 (20.0%)	20 (24.7%)
Diploma/technical	8 (26.7%)	23 (28.4%)
University graduate	9 (30.0%)	17 (21.0%)
**Household income, AUD yearly**	**N=20**	**N=54**
Less than $31,199	4 (13.3%)	9 (16.7%)
$31,200 - $77,999	9 (30.0%)	20 (37.1%)
$78,000 - $104,000	3 (10.0%)	9 (16.7%)
Over than $104,000	4 (13.3%)	16 (29.6%)
**Engagement with outreaches**	**N=30**	**N=82**
High (≥ 4 completed [a])	25 (83.3%)	51 (62.2%)
Low	5 (16.7%)	31 (37.8%)
**Engagement with website**	**N=30**	**N=82**
Visited ≥ once	19 (63.3%)	46 (56.8%)
**Outreaches delivered via conversational AI phone calls, out of seven total**	**N=30**	**N=82**
Mean, number (SD)	5.20 (1.81)	4.12 (1.94)

[a] participant answered ≥50% of questions asked in the outreach
