# Supplementary material for: Patient Perspectives on Conversational Artificial Intelligence for Atrial Fibrillation Self-Management: Qualitative Analysis
Source: J Med Internet Res. 2025 Mar 12;27:e64325. doi: 10.2196/64325 (PMC11947624; doi:10.2196/64325)
Supplement: Multimedia Appendix 3 [file jmir_v27i1e64325_app3.docx]

**Contents**

| **Table S1.** Illustrative quotes for theme: Interaction with a voice-based conversational AI program | **2** |
| --- | --- |
| **Table S2.** Illustrative quotes for theme: Engagement is influenced by the personalisation of content, delivery mode and frequency | **3** |
| **Table S3.** Illustrative quotes for theme: Improving access to AF care and information | **6** |
| **Table S4.** Illustrative quotes for theme: Empowering patients to better self-manage their AF | **8** |

**Table S1. Illustrative quotes for theme: Interaction with a voice-based conversational AI program**

| **Human-like interactions** | **Q1** – “I guess it's [phone calls] more human (…) it's a lot easier to talk” *[Participant 44, male, age 66]*  **Q2**– “(…) I suppose phone call - it’s much more personal, I guess, is the word.” *[Participant 9, female, age 54]*  **Q3** – “(…) it’s like a friend [talking] (…) giving information rather than reading stuff” *[Participant 44, male, age 66]*  **Q4** – “I suppose having someone talking to you, you probably absorb it more than if you sat down and read a brochure or something like that. So, no I didn’t mind it at all...it was fine to understand. Very clear.” *[Participant 96, female, age 50]*  **Q5** – “(…) it's sort of a two-way you interaction where there was either questions or information coming in from the other end and I was able to respond as best I could from this end (…) it's quite an interactive process (…) when someone’s speaking to you, you actually focus a little bit better. *[Participant 44, male, age 66]* |
| --- | --- |
| **Restriction to prespecified responses** | **Q6** – “there were instances where I would have liked to maybe explain the answer or to elaborate (…). [Some questions] were a little confined, a little restricted with some of the responses. (…) forcing you into a “yes” or “no” (…). A little bit of elaboration (…) might have been able to give a bit more richness to the responses (...)” *[Participant 44, male, 66]*  **Q7** – “ [I would have preferred] a free format (…). Some people might have struggled with picking a precise answer (…)” *[Participant 38, male, 54]*  **Q8** – “I did get a [call back] phone call from answering one of those questions because of the poor choice of [response options] that didn’t really suit either/or and just picked one and I got a [follow-up] phone call because of it (…)” *[Participant 20, female, 63]* |
| **Trustworthiness of hospital-delivered conversational AI** | **Q9** – “it's [AI technology] going to be the way of the future (…) we'll be talking to machines and robots (…) I was quite comfortable listening and talking (…) I could understand maybe some people are a little bit suspicious [phone calls] (…) I got stung many years ago in marketing research (…) if people had some bad experiences, they might be a bit wary [of AI calls]” *[Participant 44, male, age 66]*  **Q10** – “It [AI technology] might be a scam [laughs] (…), [I thought] originally (…), until I realised (…) that it did come from Westmead Hospital (…) and it was safe (…)” *[Participant 37, female, age 62]* |

**Table S2. Illustrative quotes for theme: Engagement is influenced by the personalisation of content, delivery mode and frequency**

| **Tailoring to own health context** | **Q1** – “I’m not currently in AF (…) I haven’t been for over twelve months. (...) [The program would be] definitely worth it if I was (…) symptomatic (…) [and] living with it 24/7. That chaos in your chest, it’s pretty awful.” *[Participant 38, male, age 54]*  **Q2** – “there’s 100 different types of AF so what’s not relevant for me is relevant for someone else (…) I was never really asked personally about my particular AF and how it happens or why it happens (…)” *[Participant 21, male, age 54]*  **Q3** – “[I] don’t live with it all the time (…) maybe a little advice targeted [to me] (…) a lot of the questions were (…) “How was your AF in the last month or so?” (…). I haven’t had AF for the last month” *[Participant 26, male, age 53]*  **Q4** – “[there’s a need to] tailor the program (…) if a person’s just been found has AF, they’re probably in a critical sort of support position in comparison [to] if they had it for five years, then I think you need a lesser support system in place (…) a phone call once a month or once every two months for them. And ongoing from there if they wish to participate beyond that. (...) If somebody moves to a different classification (…) move them into the more advanced or the more stable program” *[Participant 65, male, age 79]*  **Q5** – “I’ve had this for over 20 years so I guess it’s something that I’ve learnt to roll (…) But if I had just been diagnosed in the last year or so, I’m sure it would be more informative (…)” *[Participant 7, male, age 71]*  **Q6** – “I’m someone who’s fairly well on top of things – I research things -and I read (…) I’m on top of all the information I need for my condition (…) it didn’t add too much for me (…) [I] see the benefits for other people (…) older people or people that don’t have the smarts to seek out the information (…). It was comforting to know that the information was right (…) So, I guess, it reinforced a couple of things.” *[Participant 26, male, age 53]*  **Q7** – “I’ve had AF for quite a long time and I’ve got into it fairly well with [doctor’s name] (…) it’s just a confirmation of information I already had. *[Participant 51, male, age 77]*  **Q8 –** “I’m still in the dark about pacemakers (…) no one can tell me if I go to the beach whether I can actually dive into the water (…) if I went to the gym how much weight [I can carry] (…) [I’m] scared sometimes even to carry groceries over my shoulder where the wire is (…). What your program did beautifully- filled in a lot of the gaps and gave people background and information (…) *[Participant 44, male, age 66]*  **Q9** – “I’ve just knocked back an invite to go to America next year (…) I’ve got all this electrical heart stuff – pacemaker and AF (…) [I’m] worried about getting COVID (…) [need information about] to what extent is AF one of these underlying health issues that we hear about when we’re hearing about people dying from COVID.” *[Participant 44, male, age 66]*  **Q10** – “the sort of things they were recommending, was exercise, diet, alcohol consumption (…) smoking (…) I don’t smoke (…) [it] wasn’t applicable to me (…) it was fairly standard (…)” *[Participant 20, female, age 63]*  **Q11** – “I appreciate that probably not everyone takes their medication and knocks some (…) drinks (…) smokes (…) But I just found that some of the tips didn’t apply to me (…). [I would have liked] more relevant tips for people who are more generally taking their medication and not drinking coffee all the time.” *[Participant 44, male, age 66]*  **Q12** – “when you’re talking about (…) have a good diet (…) maybe make more suggestions along the lines of what sort of things to eat (…) there’s so many differing opinions on what’s good and what’s bad (…) give people a basic outline. (...) maybe include some dietitian-type stuff in there that would be helpful (…)” *[Participant 20, female, age 63]* |
| --- | --- |
| **Interest in novel information regarding health** | **Q13** – “A lot of stuff was repeated (…) sometimes I’d see, oh it’s the same thing, yeah so I’d just delete the message (...)” *[Participant 20, female, age 63]*  **Q14** – “I also get a lot of information from the Heart Foundation. So, a lot of things you’re sending is just doubling up” *[Participant 64, male, age 75]*  **Q15** – “I thought the reminders to go there [the website] were because there’s some new stuff (…) it seemed to be the stuff that I looked at before” *[Participant 44, male, age 66]* |
| **Flexibility provided by multichannel delivery** | **Q16** – “I think it had contacted me (…) when I was doing a walk (…) you didn't have to stop and dedicate yourself (…) I found it quite convenient (…) [phone calls are] always easier (…) for people who are (…) technology-averse or a bit older probably (…) to talk out their answers (…) I guess it's more human (…), it's a lot easier to talk (…) than it is to write” *[Participant 44, male, age 66]*  **Q17** – “I think there are some people that (…) [would] be happier just talking their answers than having to write down responses or even reading stuff. (…) to be told things is a more helpful way of gleaning the information (…) it’s like a friend [talking] (…) giving information rather than reading stuff. (…) more senior people (…) might not like to read or they might have difficulty reading if their eyesight’s not great (…) But to listen, as long as you can hear okay (…) it was an easy and convenient way of participating (…) and collecting all the tips (…)” *[Participant 44, male, age 66]*  **Q18** – “Just to be asked questions (…) it's sort of a two-way you interaction where there was either questions or information coming in from the other end and I was able to respond as best I could from this end (…) it's quite an interactive process (…) the phone calls were the best (…) you’d listen more (…) I skimmed a lot of the survey (...) but when someone’s speaking to you, you actually focus a little bit better. *[Participant 44, male, age 66]*  **Q19** – “I reckon it's better because you’re understanding what's coming out of the talk, and you can visualise something. But when I read it (…) I might think something a little bit different to what the voice says” *[Participant 11, male, age 55]*  **Q20** – “I don’t mind the surveys because you can (…) think about it (…) some of [the survey questions were] straightforward (…) others you got to give a bit of thought to it.” *[Participant 95, male, age 74]*  **Q21** – “sometimes I don’t answer the phone. Some of those times [referring to phone calls] I was at work (…). I think the surveys were better.” *[Participant 53, female, age 69]*  **Q22** – “Surveys were probably convenient because I was working from home (…) it was easier for me to check my emails, and do it when it was a convenient time. (…) if you take a call (…) you could be in a meeting (…) you’re not able to returns calls and forget or whatever. Whereas the surveys just sat in your inbox (…) I could do it (…) when I had a break” *[Participant 89, female, age 51]*  **Q23** – “my phone [signal] dropped out [during the call] (…) where I live, it’s notorious for dropping out (…) with the survey (…) there was no stoppages” *[Participant 51, male, age 76]* |
| **Overwhelmed with large volumes of information** | **Q24 –** “Every week I was receiving something - that was a bit over the top (…) Because I work, and they’d always come in the middle of the day when I was busy (...) the messages a little less frequently.” *[Participant 20, female, age 63]*  **Q25 –** “I’d like [you] to keep in touch with me and (…) if there’s issues along the way I’ll always let you know (…). Probably a few less phone calls” *[Participant 11, male, age 55]*  **Q26 –** “my main priority at the time was (…) recovering from the encephalitis (…) herpes virus (…) that took up 99 percent of [time].” *[Participant 13, female, age 67]*  **Q27 –** “I didn’t want to be reminded of (…) what I’ve got. I’m trying to stay positive. (…) I got a good specialist (…) everything I’ve need I’ve had explained to me (…) I [could] walk around (…) feeling sorry for myself (…) but I don’t do that.” *[Participant 48, male, age 80]* |

**Table S3. Illustrative quotes for theme: Improving access to AF care and information**

| **Continuity in support** | **Q1 –** “I think any program that picks up on people not being right (…) the sort of great duty of care (…) to respond the way you did [follow-up calls after alerts] (…) very commendable (…) It shows that you're more interested in the people (…) that's a good connection to build” *[Participant 44, male, age 66]*  **Q2 –** “I think the scariest bit is having an [AF] event and 24 hours later, finding yourself at home and not really knowing what happened (…) the quicker that this sort of information can be offered to people (…) the things we can do on our own, I think really helps.” *[Participant 89, female, age 51]*  **Q3 –** “you have the cardioversion and everyone’s fussing over you [in ED] (…). [Then] you’re home [by] evening. It’s sort of a whirlwind experience (…) you do speak to your cardiologist [but] there’s no real support. You’re just sent off into the world with medication (…) I found that quite daunting (…). Education sometimes gets forgotten (…) the survey was excellent in that way (…) it really sort of explained things (…). [At home] you don’t really know what you can do and you feel a bit helpless” *[Participant 89, female, age 51]*  **Q4 –** “to still be in contact with the hospital, [the hospital to] know [how] I’m doing (…) I suppose it sounds so immature. But someone cares enough to contact you! [Laughs]” *[Participant 97, female, 70]*  **Q5 –** “It has been difficult to get to a doctor (…) during COVID time you had no support from the doctors (…) you have to just look after yourself. (…) I do think the follow-up phone call and the robot thing's [conversational AI call was] good, especially when you're on your own. Just to check-in that you're on the right track” *[Participant 37, female, age 62]*  **Q6 –** “someone who didn’t have the support (…) I think just the regular phone calls and text messages are pretty important. Particularly through something like COVID. (…) someone who doesn’t have family or friends nearby (…) pretty important service” *[Participant 89, female, age 51]*  **Q7 –** “it was really good having that touch base because (…) your situation changes throughout time. Like how I answered questions prior to this ablation and after the ablation.” *[Participant 89, female, age 51]* |
| --- | --- |
| **Enhancing access to health-related information** | **Q8 –** “it [the program] was excellent (…) everyday questions don’t happen between your visits. (…) it sort of helps if we can understand what it’s all about (…) we can’t really rely on even medical practices anymore because the fact that they’re just time poor (…) I think the things can do on our own, I think really helps.” *[Participant 89, female, age 51]*  **Q9** – “I don’t know if the GP necessarily has the answers or even interested in giving too much advice (…) maybe by fear of contradicting [the cardiologist] (…) you’re only seeing a cardiologist once every six months (…) they’re so time poor that they’re doing the basic stuff (…) you really refer to the GP as more of a maintenance program and I don’t know if that was necessarily provided (…). The surveys [addressed] that gap (…). That constant communication (…) that knowledge [provided by the program], it was good” *[Participant 89, female, age 51]*  **Q10** – “going to the cardiologist usually costs you like 250 bucks (…) some people would probably not want to do that (…) If they had access to a GP (…) that answers a lot of queries (…) [but] you’re talking about some people in remote areas not everyone lives in a capital city (...) Different mobility levels (…) lots of comorbidities (…) people not very mobile (…) seeking help is hard so there's all of that to consider.” *[Participant 38, male, 53]*  **Q11 –** “the way that those little videos and explanations were really easy to understand (…) sometimes when you are in hospital and the doctors are speaking, and you’re already stressed (…) they can use terminology that’s not always easy for people to understand (…) you can’t just take it all in (…) after the event, you’ve forgotten everything that was just said to you! [Laughs] (…) making things in layman’s terms? I think really really helps.” *[Participant 89, female, age 51]*  **Q12 –** “I thought the educational resources were useful (…) informative (…) relevant; they were quite short and picky (…) I did learn a lot about it, which was helpful. (...) I thought the videos were quite easy to digest” *[Participant 44, male, age 66]*  **Q13 –** “I think before this program, a lot of this stuff about AF, I was in the dark. (...) But you’ve filled the AF gap in terms of knowledge.” *[Participant 44, male, age 66]*  **Q14 –** “I think I’m just a lot more aware now about AF and how to explain it to other people (…) I’m now probably more educated about it” *[Participant 96, female, age 50]* |

**Table S4. Illustrative quotes for theme: Empowering patients to better self-manage their AF**

| **Encouraging healthy habits through frequent reminders** | **Q1 –** “[The program] really got me on the right road and track (…). At the beginning when I started it, I didn't know which way to go (…) you hear this and you hear that. You go to your GP and he’ll say one thing and then I think I met you (…) ever since I started this program here, you know, it’s lifted me up.” *[Participant 11, male, age 55]*  **Q2 –** “Reinforcing what you’re doing if you’re doing it. And probably encouraging you to do it if you’re not doing it (…) the style and content of the text messages was quite good.” *[Participant 38, male, age 53]*  **Q3 –** “[Tthe program] changed my alcohol intake! (…) [there was] lots of information about the alcohol consumption (…) when I first went on the program I decided to myself, well I’m going to cut it back and then I thought, well, that’s no good, I must give it up, so I did. I gave up alcohol.” *[Participant 4, male, age 69]*  **Q4 –** “I’ve got my weight down (…) I saw you – I was 86.4 and today I’m about 79. (…) [the] reminders and information that came through (…) It helped with diet (…), I’ve cut out having bacon and eggs on a Sunday [laughs]! *[Participant 11, male, age 55]*  **Q5 –** “[the program] reminded me to actually settle down and monitor [myself](…) keep up my medication, keep hydrated.” *[Participant 17, male, age 40]*  **Q6** – “you have some messages regarding food. It helped me (…) follow my diet. Sometimes you cannot (…) prevent (…) [eating] those- good foods. [Laughs] But with your constant reminder it was good!” *[Participant 31, male, age 79]* |
| --- | --- |
| **Reassurance from rhythm monitoring devices** | **Q7 –** “it’s often not knowing if I’ve got it [AF] or not (…) that can cause a bit of anxiety (…) I was recently diagnosed with um moderate levels of depression and anxiety (…) from just having- getting AF every now and then. (...) So, in January I had a (…) [loop] recorder put in (…). If I feel I might be in AF, they can check it. (…) I talk to them- occasionally when I’m a bit worried” *[Participant 26, male, age 53]*  **Q8 –** “It’s a bit of a safety thing [having the AliveCor Kardia device and app] (…) it’s reassuring if you’re feel like you’re having (…) ectopic beats (…) it can sort of say it’s a reasonably normal rhythm. (…) it is reassuring if you’ve got access to that (…) you can at least get a bit of a test happening.” *[Participant 44, male, age 66]*  **Q9 –** “I live alone. And I was always frightened [of being in AF] (…) best thing I did was buy an Apple Watch (…) that kept me out of hospital. (...) It can detect AF (…) I can check my blood pressure and take my readings and then decide (…) if I have to go to hospital or not. Or try and get myself out of it. (…) I've shown my cardiologist and he's happy with me because I know how to treat it (…) if I get AF and my watch alerts me, I just get Powerade or Gatorade, a banana (…) come to bed (…) which stopped me from going to hospital. (...) [before] I’d [go] to emergency.” *[Participant 37, female, age 62]*  **Q10 –** “Occasionally, [I’d] go to the local medical clinic to see if I was in AF or not (…) But now I’ve got the loop recorder. I feel like I’m in safe hands. I know that if I do go into AF (…) they’ll be on the phone with me pretty quickly. I don’t have to guess now (…) I can relax (…) they do call you if there’s something unusual.”*[Participant 26, male, age 53]*  **Q11 –** “It would be nice to have more contact with somebody in charge of the loop controller (…) To see if it was working. If I had any problems (…) to have some kind of knowledge of (…) what your heart’s been doing for the past month (…) So I can look back and see if there is any triggers. (…) on Saturday I had no triggers. (…) [ate] salmon and salad meal the night before. Didn’t have any alcohol. (…) I couldn’t work out why (…) [maybe] I didn’t sleep very well and I’m tired and that brought it on?” *[Participant 37, female, age 62]*  **Q12 –** “I have heart monitoring at home with the pacemaker. I’ve been in to get the pacemaker checked and they've said to me, “Oh, you know you've had episodes” (…) I once said, “(…) I'm getting home monitoring. Why didn't someone contact me” (…) [device clinic said] “Oh, we probably didn't think it was important enough.” (…) that worried me afterwards (…) I think to have some support (…) [someone to say] “we’ve been picking up a bit of a few minor things you might need to walk in to see [doctor’s name].” (…) That would have been more reassuring. (…) I was worried about the wires being pulled out (…) I phoned up the number [device monitoring clinic] (…) And they said, “Oh, the readings are great. They’re fine. Good signal.” (…) I came into where the monitor was and it wasn't plugged in. (…) Someone’s telling me that the signals great (…) any instances where people can get some support or feel supported in a program is reassuring for them (…) I'm often wondering now with this heart monitoring whether they'll only take notice if I have a bloody cardiac arrest or (…) [if] my heart stops completely (…) I'd just like to be reassured if there was a glitch (…) I'm still not 100% confident in the home monitoring (…) someone there is saying, “Well, that's not important enough for us to let you know about it.” That to me is a worry because if there are things going on I'd rather know (…) even if they contact me, I feel reassured. (…) in your study you called respondents back when they said they weren't feeling great you were onto it just to check and see how they were. I think that's terrific. And I know people are busy (…) there's lots of demands on the health system. But I think to have that reassurance is important.” *[Participant 44, male, age 66]* |
